# Prognostic Value of Small Coved‐Type ST‐Segment Area in Patients With Spontaneous Type 1 Brugada Syndrome

**DOI:** 10.1111/jce.70305

**Published:** 2026-03-02

**Authors:** Nario Sano, Tetsuji Shinohara, Keisuke Yonezu, Masaki Takahashi, Hirochika Yamasaki, Kei Hirota, Hidekazu Kondo, Akira Fukui, Hidefumi Akioka, Yasushi Teshima, Naohiko Takahashi

**Affiliations:** ^1^ Department of Cardiology and Clinical Examination, Faculty of Medicine Oita University Oita Japan

**Keywords:** Brugada syndrome, electrocardiographic marker, risk stratification, ST‐segment area, ventricular fibrillation

## Abstract

**Background:**

Risk stratification in Brugada syndrome (BrS) remains challenging. Although spontaneous Type 1 ECG and clinical history (e.g., prior ventricular fibrillation [VF] or syncope) are established risk markers, additional electrocardiographic predictors are needed to refine prognostication.

**Methods:**

We retrospectively analyzed 82 consecutive BrS patients referred to Oita University Hospital. After excluding 20 patients with drug‐induced and 2 with fever‐induced Type 1 ECG, 60 patients with spontaneous Type 1 Brugada ECG were included. The maximum coved‐type ST‐segment area (mV·ms) was quantified across leads V1–V3 at standard and high‐intercostal positions. Patients were categorized into VF‐occurrence (*n* = 24) and non‐occurrence (*n* = 36) groups.

**Results:**

Over a mean follow‐up of 67 ± 59 months, 24 patients (40%) experienced VF. The maximum coved‐type ST‐segment area was significantly smaller in the VF‐occurrence group compared with the non‐occurrence group (27.7 ± 14.8 vs. 37.5 ± 16.7 mV·ms; *p* = 0.022). In multivariate analysis, inferolateral J waves (OR 11.1; 95% CI 2.85–42.8; *p* < 0.001) and a small maximum coved‐type ST‐segment area (OR 5.47; 95% CI 1.17–25.7; *p* = 0.03) were independent predictors of VF. Kaplan–Meier analysis showed significantly lower VF‐free survival in patients with a small maximum coved‐type ST‐segment area (*p* = 0.034).

**Conclusions:**

In patients with spontaneous Type 1 BrS, a small coved‐type ST‐segment area may be independently associated with VF and may represent a potential electrocardiographic marker for risk stratification.

## Graphical abstract

Electrical and structural abnormalities in Brugada syndrome lead to a smaller coved‐type ST‐segment area. A reduced ST‐segment area may identify patients at elevated risk of ventricular fibrillation.

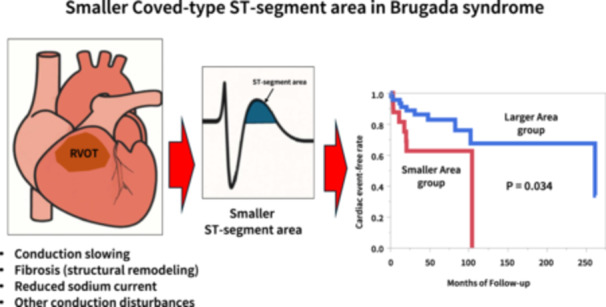

**Abbreviations**
AF: atrial fibrillation
AUC: area under the curve
CA: cardiac arrest
CI: confidence interval
ECG: electrocardiogram
ICD: implantable cardioverter defibrillator
OR: odds ratio
ROC: receiver‐operating characteristic
RVOT: right ventricular outflow tract
SCD: sudden cardiac death
SD: standard deviation
VF: ventricular fibrillation
VT: ventricular tachycardia

## 1 Introduction

Brugada syndrome (BrS) is an inherited arrhythmia disorder characterized by coved‐type ST‐segment elevation in the right precordial leads and is associated with an increased risk of sudden cardiac death (SCD) due to ventricular fibrillation (VF) [1, 2]. Despite advances in diagnosis and management, risk stratification in BrS remains challenging. Current guidelines identify prior VF, syncope, and spontaneous Type 1 ECG as reliable markers [3, 4]; however, these criteria remain insufficient to identify all high‐risk patients.

Several electrocardiographic markers—prolonged QRS duration, fragmented QRS, late potentials, and inferolateral J waves—have been proposed [5, 6, 7]. Low‐voltage phenotypes have also been linked to higher VF risk [8]. Prior studies have examined ST elevation magnitude, but systematic quantification of the hallmark coved‐type ST‐segment morphology is limited. Extramiana et al. demonstrated that quantitative assessment of ST‐segment elevation revealed dynamic variability influenced by autonomic tone [9]. Makimoto et al. reported that augmented ST elevation during the recovery phase of exercise testing predicted arrhythmic events [10]. Moreover, Rollin et al. demonstrated that the presence of Type 1 ST elevation in peripheral leads had prognostic relevance [11]. These findings indicate that the magnitude and distribution of ST elevation represent important risk markers; however, systematic quantification of the hallmark coved‐type morphology itself remains limited.

Therefore, in this study, we evaluated the prognostic significance of the coved‐type ST‐segment area—defined as the integrated value (mV·ms) from the J point to the downslope baseline—in patients with spontaneous Type 1 Brugada ECG, and investigated whether this quantitative parameter can further improve the accuracy of risk stratification in BrS.

## 2 Methods

### 2.1 Study Population

**Figure 1:**
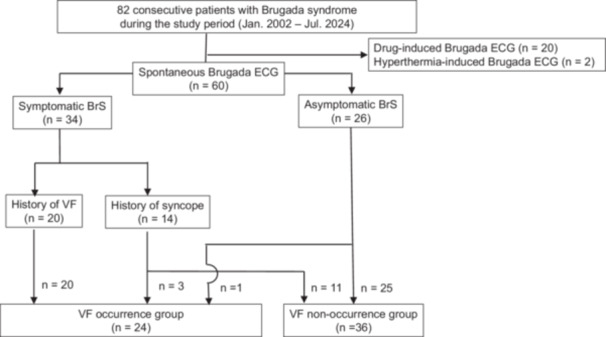
Study population and patient flow. From 82 consecutive Brugada syndrome patients, 20 with drug‐induced and 2 with fever‐induced Type 1 ECG were excluded, leaving 60 patients with spontaneous Type 1 ECG. Patients were further stratified into VF occurrence (*n* = 24) and VF non‐occurrence (*n* = 36) groups.

Between January 2002 and July 2024, 82 consecutive BrS patients referred to Oita University Hospital were retrospectively reviewed. These patients represented the same cohort as reported in our previous publication [12]. The patients were probands from 82 families who were followed for more than 1 year. The diagnosis of BrS was established according to the 2022 European Society of Cardiology (ESC) guidelines for the management of patients with ventricular arrhythmias and the prevention of SCD [13]. All patients underwent standard and high‐intercostal (second and third intercostal spaces) ECG recordings to detect Type 1 Brugada ECG. After excluding drug‐ and fever‐induced Type 1 ECGs, 60 patients with spontaneous Type 1 Brugada ECG were included (Figure 1).

At the initial visit, the cohort comprised 20 patients with a history of VF prior to enrollment, 14 patients with syncope (suspected arrhythmic syncope), and 26 asymptomatic patients. Patients were divided into two groups based on the occurrence of VF during the entire clinical course, including both prior‐to‐enrollment and follow‐up periods (VF occurrence group: *n* = 24 vs. VF non‐occurrence group: *n* = 36) for comparative analysis (Figure 1). Among the 60 patients, 27 (45%) underwent implantable cardioverter‐defibrillator (ICD) implantation; 20 for secondary prevention following documented VF and 7 for primary prevention based on clinical risk assessment. Clinical data, including medical history, laboratory findings, 12‐lead ECGs, and echocardiography, were collected from the medical records. No patient had structural heart disease based on physical examination, chest radiography, 12‐lead ECG, echocardiography, treadmill exercise ECG, Thallium‐201 cardiac scintigraphy, and coronary angiography (including coronary spasm provocation test).

This study was approved by the Oita University Faculty of Medicine Ethics Committee (Approval number: 1852). Informed consent was obtained via an opt‐out process, as approved by the ethics committee. All procedures were conducted in accordance with the principles outlined in the Declaration of Helsinki.

### 2.2 Clinical Course

Patients were followed every 3–6 months. The occurrence of VF in patients with implanted ICDs was confirmed by device interrogation. Among the 33 patients without an ICD, one experienced VF during the night, documented by an ECG obtained by paramedics. Apart from this case, no patient without an ICD experienced SCD.

### 2.3 Twelve‐Lead ECG Findings

Standard 12‐lead ECGs were recorded at rest using limb and precordial leads. All ECGs used for analysis were recorded under standardized resting conditions between 10:00 and 14:00, in order to minimize the influence of circadian variation and autonomic tone. The ECGs were obtained during routine outpatient evaluation, not specifically at the time of VF events. When multiple ECGs were available, recordings obtained under these standardized conditions and closest to the initial presentation were preferentially selected for analysis. Recordings were acquired in non‐filtered mode (0.05–150 Hz) without additional filters to avoid distortion of the ST‐segment waveform. Each ECG included simultaneous recordings (V1–V3) from standard and high‐intercostal positions (second and third intercostal spaces) in all patients. The maximum coved‐type ST‐segment area was quantified using raw, non‐filtered ECG waveforms obtained from continuous 10‐s recordings. Raw numerical waveform data (5000 samples per lead) exported from the CardiMax system (Fukuda Denshi, Tokyo, Japan) were reconstructed for ST‐segment area integration. The first and last beats of each 10‐s recording were excluded to avoid baseline instability. Beats with clear onset and termination and without artifacts were visually inspected and manually selected, and the maximum ST‐segment area among all analyzed beats was used for analysis. No corrective filtering or post‐processing was applied; all analyses were performed directly on raw ECG waveforms to ensure reproducibility.

**Figure 2:**
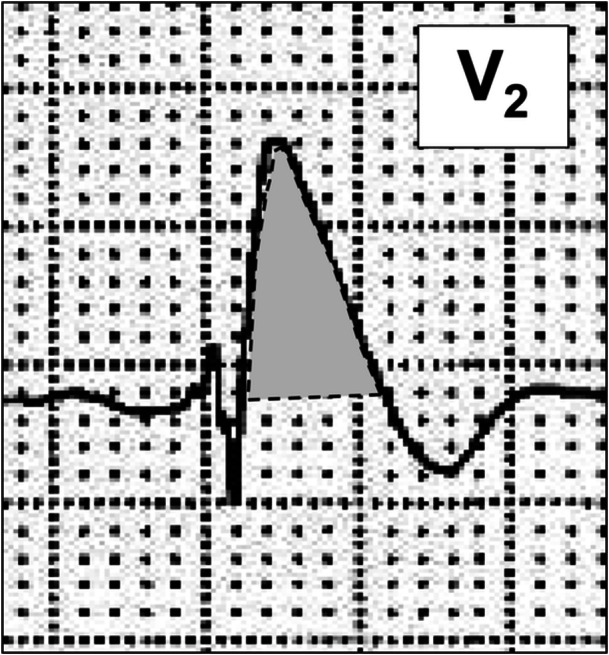
Coved‐type ST‐segment area. The shaded gray region represents the coved‐type ST‐segment area, defined as the integrated area from the J point to the baseline along the downslope of the ST segment.

**Figure 3:**
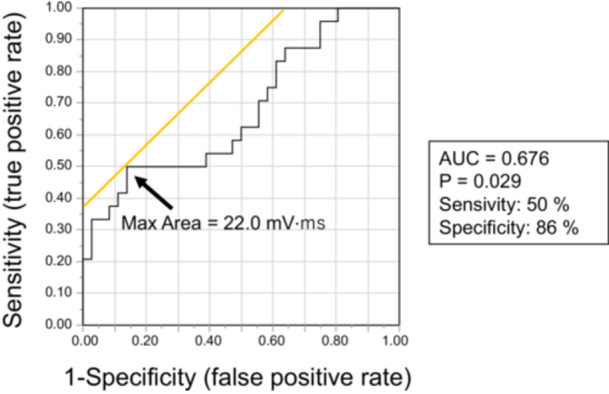
Receiver‐operating characteristic (ROC) curve analysis of maximum coved‐type ST‐segment area. A cutoff value of 22.0 mV·ms predicted VF events with an area under the curve (AUC) of 0.676 (*p* = 0.029), yielding a sensitivity of 50% and specificity of 86%.

The coved‐type ST‐segment area was defined as the integrated value from the J point to the baseline of the downslope of the ST segment, expressed in mV·ms (Figure 2). Area (mV·ms) was calculated using the trapezoidal rule. The coved‐type ST‐segment area was measured at all nine lead–intercostal combinations, and the maximum value among all recording sites was used for analysis. A cutoff value for predicting VF was determined by receiver‐operating characteristic (ROC) analysis (Figure 3). Patients were categorized into a large‐area group (≥ 22.0 mV·ms) and a small‐area group (< 22.0 mV·ms).

Late potentials on signal‐averaged ECG were considered positive when ≥ 2 of the following criteria were met: (1) filtered QRS > 105 ms; (2) RMS40 < 15 μV; (3) LAS40 > 39 ms. The r–J interval was defined as the interval from QRS onset to the J point in lead V1. T‐peak to T‐end interval was defined as the maximum T‐peak to T‐end interval in any precordial lead. The aVR sign was defined as R‐wave amplitude ≥ 0.3 mV or *R*/*q* ratio ≥ 0.75. The early repolarization pattern was defined as a “notch” or “slur” ≥ 0.1 mV in two or more inferior (II, III, aVF) or lateral leads (I, aVL, V4–V6). Fragmented QRS was defined as the presence of ≥ 1 spike within the QRS complex in two or more consecutive leads (QRS < 120 ms) [14].

### 2.4 Observer Variabilities

Intra‐observer variability was assessed by having the same observer analyze the identical 12‐lead ECGs on two separate occasions, 1 week apart. Inter‐observer variability was evaluated by a second observer, who independently reviewed the data without knowledge of the first observer's measurements (N.S. and T.S.). Both observers were blinded to the patients' clinical outcomes.

### 2.5 Statistical Analysis

Continuous variables were expressed as mean ± standard deviation (SD), and categorical variables as numbers and percentages. Fisher's exact test was used for categorical comparisons. The Shapiro–Wilk test assessed normality. Normally distributed variables were compared using the unpaired Student's *t*‐test, whereas non‐normally distributed variables were compared using the Mann–Whitney *U*‐test. Univariate logistic regression identified predictors of VF, and variables with *p* < 0.05 were entered into multivariable logistic regression to determine independent predictors. Kaplan–Meier analysis assessed VF‐free survival, and curves were compared using the log‐rank test. Model performance was evaluated using ROC analysis, and optimal cutoffs were determined using the Youden index. A *p* value < 0.05 was considered statistically significant. Statistical analyses were performed using JMP version 13.2 (SAS Institute, Cary, NC, USA).

## 3 Results

### 3.1 Patient Characteristics

During the overall clinical course, 24 patients (40%) experienced VF; 20 had a history of VF at enrollment and 4 experienced new‐onset VF during follow‐up of 67 ± 59 months. Baseline characteristics are summarized in Table 1. All patients were male, and the mean age at enrollment was 43 ± 14 years, with no significant difference between the VF occurrence and VF non‐occurrence groups. A history of VF was documented in 20 patients, all of whom later experienced recurrent VF (83% of the VF occurrence group vs. 0% of the VF non‐occurrence group, *p* < 0.001). Atrial fibrillation was more frequently observed in the VF occurrence group (13% vs. 0%, *p* = 0.03).

**Table 1:** Clinical and electrocardiographic characteristics.

	All	VF occurrence	VF non‐occurrence	*p* value
	*n* = 60	*n* = 24	*n* = 36	
Gender (male), *n* (%)	60 (100)	24 (100)	36 (100)	—
Age at enrollment (year)	43 ± 14	44 ± 14	42 ± 15	0.59
History of VF, *n* (%)	20 (33)	20 (83)	0 (0)	< 0.001
History of syncope, *n* (%)	20 (33)	9 (38)	11 (31)	0.58
Family history of SCD, *n* (%)	15 (25)	7 (29)	8 (22)	0.54
History and/or presence of AF, *n* (%)	3 (5)	3 (13)	0 (0)	0.03
Positive late potentials, *n*/*N* (%)	33 of 53 (62)	15 of 21 (71)	18 of 32 (56)	0.26
r–J interval in lead V_1_ (ms)	106 ± 17	112 ± 17	102 ± 16	0.003
T‐peak to T‐end interval (ms)	91 ± 14	94 ± 15	90 ± 13	0.14
aVR sign	8 (13)	5 (21)	3 (8)	0.16
Early repolarization pattern in inferior and/or lateral leads, *n* (%)	26 (43)	18 (75)	8 (22)	< 0.001
Fragmented QRS, *n* (%)	47 (78)	22 (92)	25 (69)	0.04
Maximum coved‐type ST‐segment area (mV·ms)	33.6 ± 16.5	27.7 ± 14.8	37.5 ± 16.7	0.022
Positive for VF induction test	11 of 45 (24)	6 of 19 (32)	5 of 26 (19)	0.34
ICD implantation, *n* (%)	27 (45)	23 (96)	4 (11)	< 0.001
Mean follow‐up periods (months)	67 ± 59	62 ± 74	71 ± 48	0.05

Abbreviations: AF, atrial fibrillation; ICD, implantable cardioverter defibrillator; SCD, sudden cardiac death; VF, ventricular fibrillation.

### 3.2 Electrocardiographic Parameters

**Figure 4:**
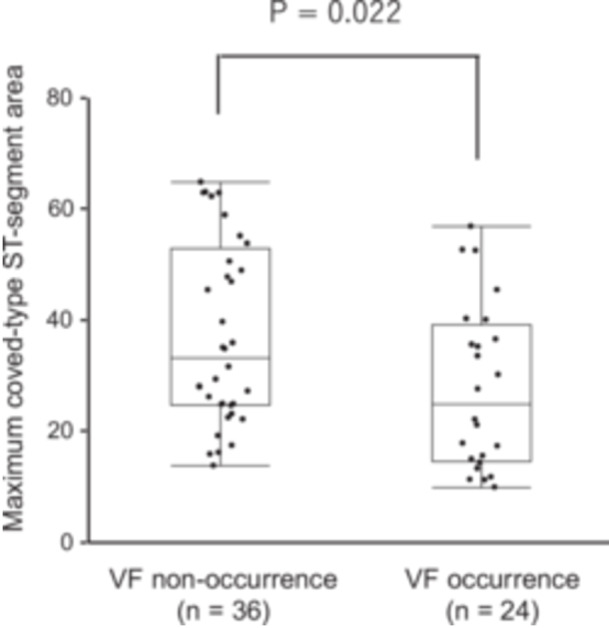
Comparison of maximum coved‐type ST‐segment area between VF occurrence and VF non‐occurrence groups. Patients with VF had significantly smaller maximum coved‐type ST‐segment area than those without VF (27.7 ± 14.8 vs. 37.5 ± 16.7 mV·ms, *p* = 0.022).

The r–J interval in lead V_1_ was significantly longer in the VF occurrence group compared with the VF non‐occurrence group (112 ± 17 ms vs. 102 ± 16 ms, *p* = 0.003; Table 1). The early repolarization pattern in the inferior and/or lateral leads was observed more frequently in the VF occurrence group (75% vs. 22%, *p* < 0.001). Fragmented QRS was also more common in the VF occurrence group (92% vs. 69%, *p* = 0.04). In contrast, the T‐peak to T‐end interval and aVR sign did not differ significantly between two groups. Notably, the maximum coved‐type ST‐segment area was significantly smaller in the VF occurrence group compared with the VF non‐occurrence group (27.7 ± 14.8 mV·ms vs. 37.5 ± 16.7 mV·ms, *p* = 0.022; Figure 4).

### 3.3 Univariate Analysis and Survival Outcomes

**Figure 5:**
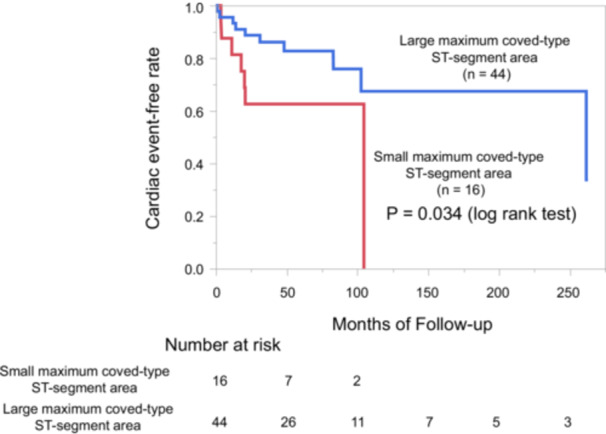
Kaplan–Meier analysis of cardiac event‐free survival according to ST‐segment area. Patients with a small maximum coved‐type ST‐segment area (< 22.0 mV·ms) showed significantly lower event‐free survival compared with those with a larger area (≥ 22.0 mV·ms; *p* = 0.034, log‐rank test).

Univariate logistic regression identified three significant predictors of VF events: early repolarization pattern in the inferior and/or lateral leads (odds ratio [OR] 10.5, 95% confidence interval [CI] 3.12–35.3, *p* < 0.001; Table 2), fragmented QRS (OR 4.84, 95% CI 0.97–24.3, *p* = 0.03), and small maximum coved‐type ST‐segment area (< 22.0 mV·ms; OR 5.25, 95% CI 1.52–18.1, *p* = 0.006). ROC curve analysis demonstrated that a cutoff value of 22.0 mV·ms for the maximum coved‐type ST‐segment area predicted VF events with an area under the curve (AUC) of 0.676 (*p* = 0.029), yielding a sensitivity of 50% and specificity of 86% (Figure 3). Kaplan–Meier curves demonstrated significantly lower VF‐free survival in the small maximum coved‐type ST‐segment area group (*p* = 0.034, log‐rank test; Figure 5).

**Table 2:** Univariate and multivariate logistic analyses of predictors for VF events.

Parameters	Univariate analysis			Multivariate analysis		
	Odds ratio	95% CI	*p* value	Odds ratio	95% CI	*p* value
Age	1.01	0.97–1.05	0.67			
Family history of SCD	1.44	0.44–4.69	0.54			
Positive late potentials	1.94	0.60–6.31	0.26			
r–J interval > 90 ms in lead V_1_	5.55	0.64–48.4	0.07			
aVR sign	2.89	0.62–13.5	0.17			
T‐peak to T‐end interval ≥ 100 ms	2.09	0.73–5.99	0.17			
Early repolarization pattern in inferior and/or lateral leads	10.5	3.12–35.3	< 0.001	11.1	2.85–42.8	< 0.001
Fragmented QRS	4.84	0.97–24.3	0.03	3.84	0.65–22.6	0.11
Small maximum coved‐type	5.25	1.52–18.1	0.006	5.47	1.17–25.7	0.03
ST‐segment area (< 22.0 mV·ms)						
Positive for VF induction test	1.94	0.49–7.66	0.34			

Abbreviations: SCD, sudden cardiac death; VF, ventricular fibrillation.

### 3.4 Multivariate Analysis

To identify independent predictors of VF events, variables that were significant in univariate analyses were entered into a multivariate logistic regression model. In multivariate analysis, early repolarization pattern in the inferior and/or lateral leads (OR 11.1; 95% CI 2.85–42.8; *p* < 0.001; Table 2) and a small maximum coved‐type ST‐segment area (OR 5.47; 95% CI 1.17–25.7; *p* = 0.03) remained independently associated with VF events.

## 4 Discussion

### 4.1 Major Findings

Three main findings emerged from the present study. First, the maximum coved‐type ST‐segment area was significantly smaller in patients who experienced VF compared with those without VF. Quantitative assessment of this ECG parameter suggested that a diminished ST‐segment area may reflect higher arrhythmic risk and may serve as a potential prognostic marker, although this finding should be interpreted cautiously. Second, both a small maximum coved‐type ST‐segment area and early repolarization pattern were identified as independent predictors of VF in multivariate analysis, underscoring the value of combining established qualitative markers with novel quantitative measures to enhance risk stratification. Third, Kaplan–Meier analysis showed that patients with small maximum coved‐type ST‐segment area had a significantly higher incidence of adverse events.

Collectively, these results support the prognostic value of the maximum coved‐type ST‐segment area. To our knowledge, this is the first study to quantitatively evaluate the coved‐type ST‐segment area and demonstrate its prognostic significance, a novel marker for risk stratification in BrS.

### 4.2 Pathophysiological Mechanisms

Our findings are consistent with those of Nagase et al. [8], who reported that low‐voltage Type 1 ECGs are associated with an increased risk of VF. A small coved‐type ST‐segment area likely reflects advanced conduction delay and structural abnormalities (e.g., fibrosis, fatty infiltration, or microstructural disarray) within the right ventricular outflow tract (RVOT). These abnormalities reduce QRS and ST amplitudes and promote conduction heterogeneity, which predisposes to VF. Furthermore, a smaller coved‐type ST‐segment area may represent the electrocardiographic manifestation of low‐voltage regions that comprehensively reflect myocardial fibrosis and conduction delay. Epicardial mapping studies have demonstrated low‐voltage and fractionated potential areas in the RVOT [15, 16, 17], which may correspond to the reduced ST‐segment area on surface ECG. Thus, a smaller coved‐type ST‐segment area can be interpreted as a quantitative indicator of substrate extent and disease severity.

In addition to conduction abnormalities, attenuation of the ST‐segment area may also indicate reduced transmural voltage gradients, in line with the repolarization hypothesis of BrS [3, 18]. Loss of the epicardial action potential dome or heterogeneous shortening of action potential duration may reduce the amplitude of ST‐segment elevation while simultaneously increasing the spatial dispersion of repolarization. This dispersion is a well‐recognized mechanism promoting Phase 2 reentry, a trigger for VF initiation [18, 19]. Importantly, the impact of repolarization abnormalities depends on their spatial distribution. Homogeneous changes may augment transmural voltage gradients and lead to prominent ST‐segment elevation. By contrast, heterogeneous changes may locally attenuate ST‐segment amplitude, resulting in a smaller coved‐type ST‐segment area. Taken together, a small coved‐type ST‐segment area likely reflects the combined effects of conduction slowing and repolarization heterogeneity.

Thus, the ST‐segment area appears to integrate the effects of both depolarization and repolarization abnormalities, effectively bridging the two major mechanistic theories of BrS. Unlike conventional indices that rely solely on amplitude or morphology, quantitative assessment of the ST‐segment area incorporates both voltage and temporal components. This comprehensive approach may provide a more accurate representation of the arrhythmogenic substrate, thereby yielding novel mechanistic and prognostic insights.

### 4.3 Clinical Implications

From a clinical perspective, the quantitative assessment of the ST‐segment area offers several advantages. It can be derived from standard 12‐lead ECGs without the need for specialized equipment, ensuring broad applicability in routine practice. Importantly, its prognostic value is independent of established risk markers such as syncope, prior VF, and spontaneous Type 1 ECG, indicating that it may complement and refine current risk stratification strategies [20, 21, 22].

Incorporating the ST‐segment area into multiparametric risk models may help identify high‐risk individuals who would otherwise be overlooked. This could guide more appropriate patient selection for ICD therapy and improve individualized management of BrS [23, 24, 25].

### 4.4 Limitations

Several limitations should be acknowledged. First, this was a retrospective single‐center study with a relatively small sample size, which may limit the generalizability of the findings. Second, although the ST‐segment area was quantitatively measured, intra‐ and inter‐observer variability was not comprehensively assessed. Third, the cutoff value derived from ROC analysis demonstrated only modest sensitivity, and external validation in larger, prospective, multicenter cohorts will be required to confirm its robustness. Fourth, genetic testing was not uniformly performed, and therefore potential genotype–phenotype interactions influencing the ST‐segment area could not be assessed. Finally, other clinical and electrophysiological variables such as autonomic tone, medication use, and circadian variation were not fully accounted for, which may have influenced the associations.

## 5 Conclusion

A small maximum coved‐type ST‐segment area appears to be associated with VF risk in patients with spontaneous Type 1 Brugada ECG. This simple, quantitative ECG parameter may serve as a practical tool to improve risk stratification in BrS.

## Ethics Statement

The study was approved by the ethics committee of Oita University Faculty of Medicine (Approval No. 1852).

## Consent

Informed consent was obtained using an opt‐out approach approved by the ethics committee.

## Conflicts of Interest

The authors declare no conflicts of interest.

## Data Availability

The data underlying this article will be shared on reasonable request to the corresponding author.
